# Effects of a Nurse–Manager Dualistic Intervention (NMDI) Program on Work Engagement and Job Crafting of ICU Burnout Nurses: A Quasi-Experimental Study

**DOI:** 10.1155/2024/6828123

**Published:** 2024-10-28

**Authors:** Fang-Yan Yue, Si-Jia Wang, Yun Du, Feng-Ye Sun, Yu-Ping Wang, Yu-Fang Guo

**Affiliations:** ^1^School of Nursing and Rehabilitation, Shandong University, Jinan, Shandong, China; ^2^Shandong Provincial Hospital Affiliated to Shandong First Medical University, Jinan, Shandong, China

**Keywords:** burnout, job crafting, nurse, work engagement

## Abstract

**Objective:** To assess the effects of the nurse–manager dualistic intervention (NMDI) program on work engagement and job crafting of ICU burnout nurses.

**Background:** Work engagement is crucial for nurses' job performance and quality of clinical care. Personal and work resources are important antecedents of work engagement. However, few intervention studies focused on improving nurses' personal and work resources to promote work engagement and job crafting of burnout nurses.

**Methods:** This was a quasi-experimental study. One hundred and two ICU nurses from two tertiary hospitals in Shandong Province were recruited. Forty-two ICU nurses from one hospital were set as the intervention group and underwent NMDI. Sixty ICU nurses from the other hospital constituted the control group, which received routine occupational health guidance from the hospital. Demographic characteristics, burnout, work engagement, and job crafting were collected at baseline (T0), postintervention (T1), and 3-month postintervention (T2).

**Results:** Compared to baseline, both work engagement and job crafting scores increased in the intervention group at postintervention (T1). At postintervention (T1), work engagement and job crafting were significantly higher in the intervention group than in the control group (*β*_workengagement_=3.894, *p*=0.016 and *β*_jobcrafting_=6.104, *p*=0.010), but the difference between the two groups was not significant at the 3-month follow-up (*β*_workengagement_=3.618, *p*=0.066 and *β*_jobcrafting_=3.554, *p*=0.15).

**Conclusion:** The NMDI program can significantly improve ICU burnout nurses' work engagement and job crafting. Nevertheless, the sustainability of these effects over time has been found to be limited, indicating that future research needs to explore and implement strategies to bolster the long-term efficacy of this intervention.

**Implications for Nursing Management:** Nurse managers are suggested to integrate the NMDI program into routine nursing management. Managers ought to prioritize appreciative and constructive dialog between themselves and nurses in order to support nurses in inquiring personal and work resources and encourage nurses to develop work plans to utilize resources. This will help to increase nurses' engagement and job crafting.

**Trial Registration:** ClinicalTrials.gov identifier: ChiCTR2100047974

## 1. Introduction

Work engagement is a positive and fulfilling work-related mental state marked by vigor, dedication, and absorption [1]. Nurses who exhibit high levels of energy and work engagement have superior job performance and elevated levels of overall well-being [2]. Studies showed that work engagement coexists with burnout, and these two phenomena exhibit a noteworthy and distinct inverse relationship [3]. Nurses experiencing heightened burnout levels tend to display diminished work engagement [4], which can potentially heighten the likelihood of medical errors and adverse events for patients [5], impair nurses' job performance, escalate turnover rates [6], and adversely affect health and well-being of nurses themselves [7]. ICU nurses are one of the groups of nurses who have a higher risk of burnout compared to other general unit nurses because of higher intensity, longer hours, and the need to cope with end-of-life patients, ethical decision-making, and miscommunication [8]. A national study in China showed that up to 68.3% of ICU nurses suffer from burnout [9]. Therefore, it is crucial for nurse managers to explore effective interventions to enhance the work engagement of ICU nurses experiencing burnout. This study explores a new appreciative inquiry (AI)–developed burnout mitigating intervention for ICU nurses in China based on the job demands–resources (JD–R) occupational stress model by Bakker and Demerouti [10].

## 2. Background

**Figure 1:**
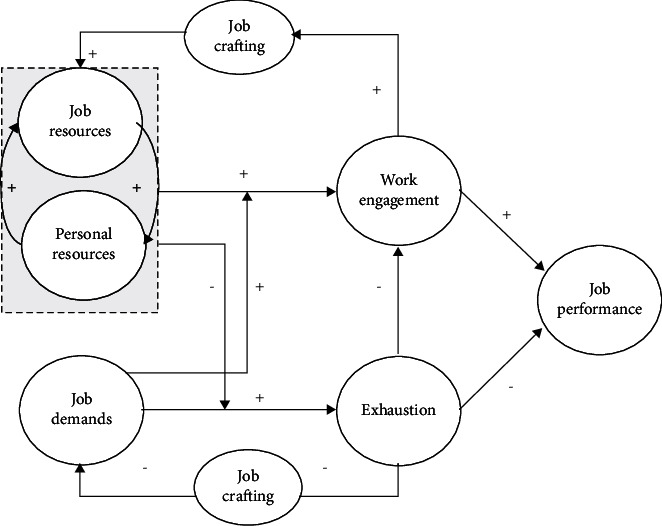
The job demands–resources model. Data from Bakker and Costa [12].

According to the JD–R model, personal and work resources are the most important predictors of work engagement [11] (see Figure 1). Job resources encompass physical, social, and organizational elements of a job, such as feedback, social support, and development opportunities. Personal resources refer to positive self-assessment and an individual's beliefs about their ability to effectively influence and manage their surroundings, including self-esteem, self-efficacy, resilience, and optimism. These resources help to alleviate the demands of the job and help employees to achieve their work goals. Job crafting refers to the proactive behavior of employees in redesigning their jobs with the aim of optimizing the match between job demands and resources [13]. The JD-R model suggests that work and personal resources activate a motivational pathway that leads to job crafting, work engagement, and better work performance [14]. In light of these insights, interventions aimed at enhancing resources have been developed, including personal resource-building and work resource-building interventions.

Personal resource-building interventions focus on increasing an individual's self-perceived positive attributes and strength, which includes strength utilization, self-resource mobilization, and career self-management. Bakker and Van Wingerden [15] encourage participants to identify, develop, and utilize their intrinsic strength to their best advantage, and the results showed a significant increase in work engagement. Coo and Salanova [16] utilized an intervention to mobilize participants' inherent energies or resources and showed that the intervention increased participants' work engagement, well-being, and performance. In the study conducted by Akkermans et al. [17], participants engaged in career development interventions to reflect on and develop their skills and competencies (e.g., self-efficacy and resilience), which had a positive impact on work engagement.

Work resource-building interventions focus on resources in the work environment, such as autonomy, social support, and feedback. It includes job crafting, leadership training, and enhanced management techniques. Gordon et al. [18] used a job crafting intervention to encourage participants to proactively change social relationships at work and design work programs. Participants' work engagement significantly increased as a result of the study. Peláez Zuberbuhler et al. [19] provided leadership training to leaders, which resulted in significant improvements in employee engagement and performance. A study conducted by Madede et al. [20] facilitated healthcare workers in receiving constructive supervision and feedback by enhancing management support mechanisms. The results showed that this intervention had a positive impact on participants' job satisfaction, burnout, and work engagement.

Numerous interventions have been developed and have demonstrated positive outcomes. However, recent systematic reviews and meta-analyses showed that these interventions had only small effects on work engagement [21, 22]. Intervention strategies that are notably effective in increasing work engagement still need to be explored. Given that both personal and work resources are important antecedents of work engagement, intervention strategies that simultaneously intervene with both resources are suggested to be the most effective approach [23]. To date, there is a lack of research on effective interventions to simultaneously improve nurses' personal and work resources in order to increase nurses' work engagement. Therefore, it is essential to validate the effectiveness of new interventions that target both aspects of resources.

The NMDI program is an intervention designed to increase both personal and work resources to enhance work engagement [24]. It was developed by our research team based on the theory of AI and the four-phase implementation process [25]. The NMDI program suggests that individuals and organizations can achieve their goals and visions by continually exploring and using their strength and resources, which fits well with the JD-R model's view of enhancing personal and work resources to promote work engagement. Therefore, the NMDI program may be a potential strategy for increasing both resources and increasing work engagement [26].

The NMDI program consists of several essential components, which include developing a positive theme, encouraging nurses to inquire about work and personal resources in conversations with their managers, creating a dream of nursing that inspires energy and enthusiasm, and developing sustainable implementation strategies [24]. Through these components, the NMDI program leads nurses and nurse managers to interact from a positive perspective rather than a problematic and inadequate perspective, which will help foster positive emotions and positive self-evaluation. In the process, nurses will inquire about, mobilize, and utilize their inherent strength and work resources in order to maximize productivity, excellence, and engagement in their work. In addition, through the process of constructive dialog between nurses and managers, a deeper understanding of their own goals and responsibilities is achieved. With this shared understanding, they develop a strong sense of cooperation and ownership of their work, which will promote job crafting behaviors.

Therefore, we hypothesized that the NMDI program would promote job crafting and work engagement among ICU burnout nurses. The purpose of this study was to assess the effects of the NMDI program on work engagement and job crafting among ICU burnout nurses.

## 3. Methods

### 3.1. Research Design

This study employs a quasi-experimental design.

### 3.2. Participants

In this study, we used convenience sampling to select two tertiary hospitals located in East China. Tertiary hospitals in mainland China are reputed as comprehensive hospitals, staffed with high-quality personnel and well-equipped facilities while providing cross-regional, high-level services, education, and research. We conducted a matching of ICU bed counts and nurse-to-patient ratios between the two hospitals to reduce biases arising from variations in hospital characteristics. The number of beds and the ratio of nurses to patients in the ICUs of the two hospitals participating in the study were comparable. Participants in the study were recruited from the ICUs of both hospitals. Participants were eligible if they met the following criteria: (i) Experienced occupational burnout, indicated by a Maslach Burnout Inventory–General Survey (MBI–GS) score of ≥ 1.5; (ii) directly involved in patient care work and had worked for at least 3 months in the past half year; and (iii) willing to participate in the study. Refresher nurses were excluded. We selected ICU nurses from one hospital as the intervention group and ICU nurses from another similar hospital were matched as the control group.

Sample calculation: G-Power 3.1 is utilized to calculate the sample size, selecting an independent samples *t*-test for mean comparison. Based on similar previous studies, the effect size of the intervention on the primary outcome measure, work engagement, is Cohen's *d* = 0.72 [27]. Therefore, the software was configured as follows: two-tailed test, *α* = 0.05, 1−*β* = 0.8, and *d* = 0.72, resulting in a minimum sample size of *n*1 = *n*2 = 32. Considering a 15% sample attrition, the intervention and control groups required a minimum of 38 participants for each group.

### 3.3. Intervention

The NMDI program was developed as an intervention guided by the framework for complex interventions proposed by the Medical Research Council (MRC) in the UK. The program was developed as an intervention based on the theory of appreciative inquiry and its 4D cycle process. It consists of 6 phases as follows: (i) Introduction: nurses become familiar with the intervention program and process; (ii) inception: identifying a common affirming theme; (iii) discovery: discovering nurses' existing work and personal resources and helping nurses to excavate potential resources; (iv) dream and design: creating a dream of nursing to make a difference and inspire the nurse's energy and passion, constructing a compelling strategy for practice and must have the essential elements of a resource for promotion; (v) destiny: practicing strategies in normal clinical work with organizational support and sharing best practice, and (vi) keep: keep sustaining in normal clinical settings and re-enquiry. The details of the NMDI program are listed in Table 1.

The NMDI program is an offline and online intervention. The offline intervention consisted of five weekly sessions (introduction, inception, discovery, dream and design, and keep), with weekly sessions lasting for 30–60 min. The intervenor implements the intervention program for each session and provides weekly supervision. In addition to the nurses in the intervention group, we invited the nurse manager to participate in each meeting. The nurse manager plays a crucial role in supporting the nurses and assisting with the implementation process in a clinical setting. Their responsibilities include identifying key topics, providing organizational resources, and supervising the design and execution of the intervention strategy. Nurses engaged in practical application and nursing managers provided guidance and oversight. The online intervention consisted of a 7-week session (destiny) that required participants to practice strategies in clinical settings. To ensure fidelity of strategy implementation and provide assistance with any difficulties encountered during implementation, the online session was delivered every two weeks with sessions lasting for 30–60 min using Tencent Conference V3.24.2 (Tencent Corporation, Shenzhen, China; a free high-definition cloud conferencing software). Participants were required to join the online meetings using their real names while turning on their cameras and maintaining a quiet surrounding environment.

The control group received routine occupational health guidance from the hospital. Specifically, all members of the control group underwent at least one face-to-face health consultation during the study period, which included advice on career development and occupational mental health guidance. These consultations were provided by psychological and occupational health experts within the hospital, with each session lasting approximately for 30 min.

### 3.4. Measurement

The self-report questionnaires were administered in a paper format at three different time points: baseline (T0), postintervention (T1), and 3 months after the intervention (T2). The collected data encompassed various aspects such as demographic information, burnout, work engagement, and job crafting.

The sociodemographic variables encompassed in the dataset consisted of age, sex, professional rank, educational background, marital status, years of service, monthly income (after taxes), presence of children, and mode of employment.

Burnout was measured using the MBI–GS [28], which comprises 16 items separated into three subscales: emotional exhaustion, cynicism, and reduced professional effectiveness. These items were scored on a seven-point Likert scale (0 = never and 6 = daily). Nurses who surpassed a burnout score of 1.5 were considered burnout [29]. The scale in this study had a Cronbach alpha coefficient of 0.86.

#### 3.4.1. Primary Outcome

In this study, we measured “work engagement” using the Utrecht Work Engagement Scale (UWES-9) in its Chinese version [30]), which consists of three dimensions, vigor (three items), dedication (three items), and absorption (three items), with a total of nine items. The scale is scored on a seven-point scale from 0 to 6, with 0 being “never” and 6 being “every day,” and the total score is the sum of each item, with higher scores indicating higher levels of work engagement. In this study, the Cronbach's alpha value is 0.816, indicating the good reliability of this method.

#### 3.4.2. Secondary Outcome

The “job crafting” variable was measured using the Chinese version of the Job Crafting Scale (JCS) [13]. The scale is based on the job demands-resources model and consists of increasing structural job resources, increasing social job resources, increasing challenging job requirements, and reducing obstructive job requirements. There are 4 dimensions and a total of 21 questions. The items are rated on a five-point scale, with 1 being “never” and five being “often.” The reliability and validity of the scale were good, with a Cronbach's alpha value of 0.924 in this study.

#### 3.4.3. Control Variables

The representative variable of family factors affecting work engagement and work–family conflict was chosen as the control variable of this study. The Work–Family Conflict Scale developed by Netemeyer et al. was used to measure the variable. The Cronbach's alpha coefficient was 0.882 in this study [31].

### 3.5. Data Collection

After obtaining permission from the hospital administrators, study recruitment flyers were distributed in the department. All nurses who expressed interest in participating in the program were introduced to the purpose and process of the study. Subsequently, eligible nurses were selected according to predetermined inclusion and exclusion criteria. Those meeting the criteria provided written informed consent prior to participation. Then, a WeChat group (Tencent Inc., Shenzhen, China) with all eligible nurses was created, through which electronic questionnaires were distributed for completion at three time points: baseline (T0), immediately postintervention (T1), and 3-month postintervention (T2).

### 3.6. Data Analysis

The data were analyzed using SPSS 27.0. Descriptive statistics, including means, standard deviations, and percentages, were utilized to describe the sociodemographic characteristics, work engagement, and job crafting. The *t*-test and *χ*-test were employed to estimate the differences between the baseline data of the two groups. The generalized estimating equation (GEE) model was used to examine the effects of intervention, time, and the interaction of intervention and time on work engagement and job crafting. Outcomes were analyzed using intention-to-treat (ITT) principles. All available data for this study were not interpolated for missing values. All participants had complete baseline data and were included in the GEE analysis. GEE was chosen due to its ability to account for internal correlations between repeated measurements and generate unbiased estimates, even when data are missing, as long as the missing data are completely randomized.

### 3.7. Ethical Considerations

Approval was obtained from the Ethics Committee of the School of Nursing and Rehabilitation, Shandong University, and the participating hospitals (no. 2019-R-011). All participants provided written informed consent. Confidentiality was assured.

## 4. Results

### 4.1. Demographic Characteristics of the Sample

**Figure 2:**
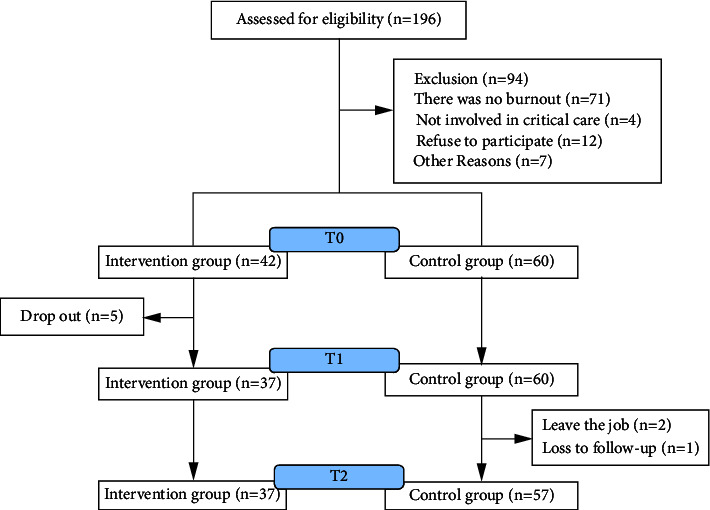
Participant flow diagram.

There were 102 nurses who participated in the study at baseline, 42 in the intervention group and 60 in the control group. After the intervention, three were lost to follow-up in the control group. Finally, 94 nurses completed the entire study, 37 from the intervention group and 57 from the control group. The flowchart of participation is presented in Figure 2.

At baseline, there were no significant differences between the intervention and control groups in sex, marital status, fertility, education, working time, or professional rank (all *p* > 0.05) (Table 2). There were also no significant differences between the two groups in burnout, work engagement, or job crafting (all *p* > 0.05) (Table 3). However, the differences between the two groups in age, mode of employment, monthly income, and work–family conflict were statistically significant (all *p* < 0.05), which were treated as covariates in subsequent analyses (Table 2).

### 4.2. Effects of Intervention on Work Engagement

GEEs were used to analyze group main effects, time main effects, and group × time interaction effects using the work engagement score as the dependent variable. Age, employment mode, monthly income, and work–family conflict score were also included in the model to control. The results showed significant group main effects (Wald chi-square = 4.584, *p*=0.033) and time main effects (Wald chi-square = 26.943, *p* < 0.001), however, nonsignificant group ∗ time interaction effects (Wald chi-square = 0.377, *p*=0.805).

Further comparisons of work engagement were made between groups at each time point as well as within-group comparisons during the assessment period. As shown in Table 4, in the intervention group, work engagement was significantly higher at postintervention (T1) and 3-month postintervention (T2) compared to baseline (T0) (*β*_*T*1_=6.12, *p* < 0.001 and *β*_*T*2_=6.27, *p*=0.001), and a significant rise was similarly observed is the control group (*β*_*T*1_=4.706, *p*=0.004 and *β*_*T*2_= 5.126, *p* < 0.001). During the between-group analysis, it was observed that work engagement exhibited a statistically significant increase in the intervention group compared to the control group at postintervention (*β* = 3.894, *p*=0.016). However, the disparity between the two groups was not deemed statistically significant at the 3-month postintervention assessment (*β* = 3.618, *p*=0.066).

### 4.3. Effects of Intervention on Job Crafting

The results of the GEEs showed a significant time main effect of job crafting (Wald chi-square = 8.349, *p*=0.015) and group × time interaction effect (Wald chi-square = 5.830, *p*=0.041), while the group main effect was not significant (Wald chi-square = 2.762, *p*=0.097).

Results of within-group comparisons showed that significant improvement was observed in the intervention group at postintervention (T1) and 3-month postintervention (T2) (*β*_*T*1_=4.764, *p*=0.005 and *β*_*T*2_=5.045, *p*=0.003). In the control group, no significant improvement was observed at both T1 and T2 (*β*_*T*1_=−1.566, *p*=0.406 and *β*_*T*2_=−1.911, *p*=0.305). In between-group comparisons, job crafting was significantly higher in the intervention group than the control group at postintervention (T1) (*β* = 6.104, *p*=0.01), but at 3-month postintervention (T2), the difference between the two groups was not significant (*β* = 3.554, *p*=0.15) (Table 4).

## 5. Discussion

The concerning issue of low work engagement among burnout nurses necessitates attention due to its undeniable negative consequences [32]. Given the unsatisfactory outcomes of current work engagement strategies [21], it is imperative to develop effective strategies to enhance work engagement in burnout nurses. This study employed the NMDI program for burnout nurses and assessed its effects on work engagement and job crafting. Results indicated that the program was effective in promoting work engagement and job crafting among burnout nurses. This study enriches existing interventions that target both resources and provides an effective approach to improving burnout nurses' work engagement and job crafting.

In this study, work engagement in the intervention group was consistently increased from baseline to postintervention. Work engagement increased significantly in the intervention group compared to the control group after the intervention. This finding underscores the significance of nursing organizations incorporating programs that address both personal and work resources concurrently in order to bolster nurses' work engagement. The NMDI program emphasizes the attainment of shared objectives through dialog, self-reflection on personal strength, exploration of work resources through interactions with managers and colleagues, and the utilization of personal and work resources in the pursuit of common goals. This improves nurses' resource pool for coping with work tasks, increases nurses' enthusiasm for their work, reduces burnout, and increases work engagement. Nielsen et al. [33] reported that personal strength and workplace resources were critical to promoting people's work engagement, performance, and well-being. Through helping people explore and utilize their personal resources and work resources, participants can be helped to overcome adversity and become more engaged in their work [34]. Furthermore, the adoption of an affirmative and positive perspective rather than working from a problematic perspective may constitute another efficacious facet of the program, which can enhance participants' enthusiasm and confidence [35]. This is considered very important as these are the key drivers of work engagement. Positive psychology intervention also showed that taking a positive perspective can increase positive emotions and promote positive behaviors, which in turn promotes work engagement [36].

The control group also had significantly higher work engagement from baseline to 3-month postintervention. This may be due to the surge in COVID-19 cases in the study sites during the two postintervention assessments. Nurses play a crucial role in the fight against this pandemic. In this case, nurses are given more material and psychological support by hospitals and society. In addition to feeling the value and significance of their work, which motivates them to be more engaged in their work [2, 37], Yang et al. [38] found that nurses involved in COVID-19 care experienced greater pride and professional fulfillment, which enhanced their professional identity, dedication, and commitment to their work.

Regarding job crafting, the study showed that job crafting in the intervention group increased significantly from baseline to postintervention. After the intervention, job crafting was significantly higher in the intervention group compared to the control group. Job crafting is the behavior of redesigning work [39]. It is important in the work of nurses as it helps nurses to maximize the use of resources and optimize the balance between resources and work demands, leading to higher levels of well-being and productivity [40]. The NMDI program requires nurses to design their own work plans and implement them based on their self-imposed work goals and the resources they inquired about. These help nurses to initiate positive changes to optimize their work methods, accomplish their work goals, and better adapt to their work. El-Gazar et al. [41] obtained similar results in their study of an intervention to promote job crafting behaviors. Increased resources and self-designed work plans are essential for promoting job crafting, work passion, and commitment.

The program was observed to be ineffective at the 3-month follow-up. Sustaining the benefits of intervention programs implemented in clinical settings has been a challenge, with many effective interventions observed to decline or disappear at follow-up [42]. This is influenced by a variety of factors such as intervention strategy and context [41, 43]. In this study, due to the extended duration of the intervention (3 months), participants may have forgotten what they learned because of the lack of intensity and repeatability. The diminishing effects of the intervention may also be associated with a decline in motivation over time, as participants may not have received sufficient reminders or support to sustain their application for the NMDI program. To enhance long-term effectiveness, implementing a refresher course after the intervention period could increase both intensity and repetition. Such a course or intensive intervention could also provide ongoing support and motivation, facilitating participants' integration of the NMDI program into real-world settings. Furthermore, it is essential to explore the potential role of web and mobile applications in enhancing the sustainability of the NMDI program's effects, given the convenience of accessing learning materials online at any time.

### 5.1. Limitations

Several limitations should be considered. First, nurse participants were recruited from two tertiary hospitals in Jinan, China, which may limit the generalizability of our findings to other countries. Furthermore, this study employed a quasi-experimental research design, which may be affected by confounding factors that compromise our interpretation of causality, such as the work environment (e.g., work culture, regulations) and patient admissions. However, it is essential to acknowledge the beneficial effects of intervention on nurses. Future studies should address these limitations and further validate the potential effects of the intervention by increasing the sample size and utilizing a randomized controlled study design.

## 6. Conclusion

This study showed that this NMDI program can improve ICU burnout nurses' work engagement and job crafting. However, the sustainability of these effects is limited, highlighting the need for further enhancements to current intervention strategies in order to optimize long-term efficacy.

## 7. Implications for Nursing Management

In practice, nursing managers should pay close attention to the job crafting and work engagement of burnout nurses, as they are closely related to work outcomes and organizational results. The results of this study suggest that the NMDI program is an effective and highly acceptable intervention to improve work engagement and job crafting. Nurse managers need to recognize the power of dialog and positive affirmation. This is important in guiding them to actively explore their self-strength and organizational resources and to use them. For example, nursing managers can incorporate the sharing of “peak experiences,” reflections by nurses on the most rewarding moments in their practice, into regular staff meetings. This practice provides an opportunity to leverage both personal strength and organizational resources. Nursing administrators are encouraged to adapt the intervention program to align with their specific culture, background, and management style, thereby enhancing its feasibility and utility. In addition, exploring further potential benefits of the NMDI program in nursing practice is recommended. The NMDI program has already demonstrated a positive impact in ICU settings, and we believe its principles offer promise for broader application across diverse healthcare environments. For instance, integrating the NMDI program into nursing education, new nurse training, and similar programs could be highly beneficial.

## Figures and Tables

**Table 1:** Titles, contents, and time of the NMDI program.

Session titles	Contents	Time
(1) Introduction phase	(1) The first meeting was organized with several icebreaker games (2) Researchers introduce the concepts, theory of AI, the process, and consideration of the intervention (3) Nurses to become proficient with each other and understand the purpose and content of the intervention	1 week
(2) Inception phase	(1) Nurses and managers work together to “brainstorm” (2) Develop affirmative themes around the overall agenda of “improving performance, personal development, and organizational advancement”	1 week
(3) Discovery phase	(1) Each nurse shares peak experience and completes a character strength survey to explore personal strength (2) Communicate with manager and coworkers to make a list of organizational resources that can be used to address work-related issues	1 week
(4) Dream and design phase	(1) Build career dreams using personal strength and organizational resources and work plans to achieve key objectives (2) Share dreams and plans with group members and manager and coworkers offer suggestions	1 week
(5) Destiny phase	(1) Implementation of the plan, in which the researcher and manager provide help and supervise participants to guarantee their performance followed the work plan (2) Meetings are organized to share positive experiences and difficulties	7 weeks
(6) Keep phase	(1) Strengthen personal and organizational resources through summaries and feedback (2) To continue setting goals and a work plan	1 week

Abbreviation: NMDI = nurse-manager dualistic intervention.

**Table 2:** The baseline comparison of sociodemographic characteristics between intervention and control groups (*n* = 102).

Items	Intervention group (*n* = 42)	Control group (*n* = 60)	*t*/*x*^2^	*p*
Burnout	39.02 ± 7.50	37.73 ± 6.71	0.911	0.365
Work–family conflict	17.52 ± 4.10	15.03 ± 3.76	3.12	**0.002**
Age (years)	29.81 ± 4.36	31.82 ± 5.40	1.997	**0.049**
Sex			1.064	0.302
Male	4 (9.5)	10 (16.7)		
Female	38 (90.5)	50 (83.3)		
Marital			0.019	0.891
Unmarried	10 (23.8)	15 (25.0)		
Married	32 (76.2)	45 (75.0)		
Fertility			1.604	0.205
Childless	13 (31.0)	26 (43.3)		
Fertile	29 (69.0)	34 (56.7)		
Education level			< 0.001	0.985
Below bachelor's degree	4 (9.5)	7 (11.7)		
Bachelor's degree and above	38 (90.5)	53 (88.3)		
Working time			1.999	0.368
< 5 years	8 (19.0)	7 (11.7)		
5∼10 years	24 (57.1)	32 (53.3)		
> 10 years	10 (23.8)	21 (35.0)		
Professional rank			1.214	0.545
Junior RN	7 (16.7)	10 (16.7)		
Senior RN	26 (61.9)	42 (70.0)		
Nurse in charge	9 (21.4)	8 (13.3)		
Mode of employment			12.921	**< 0.001**
Formally employed nurse	19 (45.2)	8 (13.3)		
Contractually employed nurse	23 (54.8)	52 (86.7)		
Income			18.275	**< 0.001**
< 5000 yuan (US, $700)	9 (8.8)	3 (7.1)	6 (10.0)	
5000–10000 yuan (US, $700-1400)	63 (61.8)	17 (40.5)	46 (76.7)	
> 10,000 yuan (US, $1400)	30 (29.4)	22 (52.4)	8 (13.3)	

*Note:* Data are expressed as *n* (%) or the mean ± standard deviation. Bold values indicate *p* < 0.05.

**Table 3:** The scores for burnout, work engagement, and job crafting of nurses (*n* = 102).

Items	Group	T0 (M ± SD)	T1 (M ± SD)	T2 (M ± SD)	*p* a
Work engagement	Intervention	24.83 ± 6.80	31.59 ± 8.05	31.49 ± 10.70	0.918
	Control	24.98 ± 7.46	29.57 ± 8.78	29.84 ± 10.52	

Job crafting	Intervention	75.43 ± 10.22	80.62 ± 10.97	81.11 ± 11.16	0.801
	Control	75.97 ± 10.82	74.25 ± 12.54	77.56 ± 14.84	

Abbreviation: SD = standard deviation.

^a^Independent samples *t*-test for intervention and control groups at T0.

**Table 4:** Results of generalized estimating equations' (GEEs) test to assess the effects of time and group on the outcome of intervention on work engagement and job crafting.

Sources	Time	Within-group difference				Between-group difference	
		Intervention		Control		Intervention (control: ref.)	
		Beta (SE)	*p*	Beta (SE)	*p*	Beta (SE)	*p*
Work engagement	T0	Ref.		Ref.		2.498 (1.729)	0.149
	T1	6.12 (1.667)	**< 0.001**	4.706 (1.628)	**0.004**	3.894 (1.614)	**0.016**
	T2	6.27 (1.965)	**0.001**	5.126 (1.473)	**< 0.001**	3.618 (1.971)	0.066

Job crafting	T0	Ref.		Ref.		0.507 (2.191)	0.817
	T1	3.679 (1.421)	**0.010**	−1.566 (1.872)	0.406	6.104 (2.37)	**0.010**
	T2	4.764 (1.680)	**0.005**	1.911 (1.863)	0.305	3.554 (2.47)	0.15

*Note:* T0, preintervention; T1, postintervention; T2, 3-month postintervention. Bold values indicate *p* < 0.05.

Abbreviation: SE = standard error.

## Data Availability

The data used to support the findings of this study are available from the corresponding author upon reasonable request.
